# A Novel Modular Bioreactor to *In Vitro* Study the Hepatic Sinusoid

**DOI:** 10.1371/journal.pone.0111864

**Published:** 2014-11-06

**Authors:** Xavi Illa, Sergi Vila, Jose Yeste, Carmen Peralta, Jordi Gracia-Sancho, Rosa Villa

**Affiliations:** 1 Institut de Microelectrònica de Barcelona, IMB-CNM (CSIC), Bellaterra, Spain; 2 CIBER-BBN, Centro de Investigación Biomédica en Red en Bioingeniería, Biomateriales y Nanomedicina (CIBER-BBN), Barcelona, Spain; 3 Barcelona Hepatic Hemodynamic Laboratory, Institut d′Investigacions Biomèdiques August Pi i Sunyer (IDIBAPS), Centro de Investigación Biomédica en Red en Enfermedades Hepáticas y Digestivas (CIBER-EHD), Barcelona, Spain; 4 IDIBAPS and CIBER-EHD, Barcelona, Spain; University of Navarra School of Medicine and Center for Applied Medical Research (CIMA), Spain

## Abstract

We describe a unique, versatile bioreactor consisting of two plates and a modified commercial porous membrane suitable for *in vitro* analysis of the liver sinusoid. The modular bioreactor allows i) excellent control of the cell seeding process; ii) cell culture under controlled shear stress stimulus, and; iii) individual analysis of each cell type upon completion of the experiment. The advantages of the bioreactor detailed here are derived from the modification of a commercial porous membrane with an elastomeric wall specifically moulded in order to define the cell culture area, to act as a gasket that will fit into the bioreactor, and to provide improved mechanical robustness. The device presented herein has been designed to simulate the *in vivo* organization of a liver sinusoid and tested by co-culturing endothelial cells (EC) and hepatic stellate cells (HSC). The results show both an optimal morphology of the endothelial cells as well as an improvement in the phenotype of stellate cells, most probably due to paracrine factors released from endothelial cells. This device is proposed as a versatile, easy-to-use co-culture system that can be applied to biomedical research of vascular systems, including the liver.

## Introduction

In recent years, a variety of *in vitro* 3D cell culture methods have been designed to study either the interaction of multiple cell types or the effect of certain drugs on tissue- or organ-specific microarchitecture [1]–[3]. Advances in microfabrication and polymer processing technologies have enabled the development of highly complex systems where a variety of cell types can be co-cultured in a controlled environment, thereby establishing a new multidisciplinary scientific field known as *organ on a chip* [4]–[11].

*Organ on a chip* devices have been developed to study the pathophysiology of a variety of organs, including lung [12], liver [13], gut [14] and kidney [15]. The main interest in developing such systems is to culture cells under real world conditions. For that, microfluidic structures allowing cell stimulation with culture media are needed. This is especially relevant when analyzing the behavior of endothelial cells which are continuously stimulated by blood flow-derived shear stress inside the human body. Therefore, shear stress must be applied over cultured endothelium in order to mimic the cell behavior in human vascular systems.

The liver, in particular, has attracted much of the research in *organ on a chip* due to its central role in drug metabolism, toxicity control, and the impact of clinical diseases [16]. In order to properly study the pathophysiology of liver diseases, the unique hepatic microcirculatory architecture should be considered. The liver sinusoid, mainly composed by endothelial cells and stellate cells, plays an essential role in most liver diseases since it represents the sieve plate by which oxygen and nutrients, but also toxicants and viruses, enter the parenchyma. In the specific scenario of liver cirrhosis, the hepatic sinusoid is considered a major contributor to the progression, aggravation, and also regression upon treatment of cirrhosis. Phenotypic changes in sinusoidal cells lead to de-regulated paracrine interactions that markedly contribute to parenchymal damage and, more importantly, determine the aggravation of cirrhosis due to the development of portal hypertension, and its complications [17]. Therefore, *organ on a chip* devices designed to mimic the liver should incorporate these two types of sinusoidal cells, and importantly culture them in a sinusoidal-like architectural distribution. More specifically, endothelial cells should be exposed to blood-flow derived shear stress, below hepatic stellate cells placed in the “Space of Disse”.

A variety of hepato-microfluidic bioreactors, mostly aimed at the study hepatocytes integrity, can be found in the literature [13], [16]. Although dynamic cultures are necessary to maintain the liver-specific functions [18], hepatocytes themselves should be protected from the direct influence of shear stress. This has traditionally been accomplished by the creation of scaffolds, by embedding cells in 3D hydrogels [19], [20], by means of nanoporous membranes [21], or by culturing them on grooved substrates [22]. Another strategy consists of maintaining the hepatocytes within cord-like microstructures that mimic the endothelial barrier [23]–[26]. Finally, more complex devices to maintain liver tissues under the optimal culture conditions have been also developed [27], [28].

However, none of the previously cited liver models have sufficient *in vivo* organization to co-culture and individually analyze sinusoidal cells, necessary to study their integrity and their paracrine communications. In the present work, a microporous membrane separates two cell culture microfluidic chambers. This permits biomechanical stimulation in the upper compartment, paracrine interactions through the membrane, and static culture in the bottom compartment. Although previous studies have used a similar design [12], [15], [29]–[32], in those studies the membrane was glued to the bioreactor, resulting in a unique and compact device that compromises the individual analysis of the different cell types after being co-cultured. Moreover, in the aforementioned systems, cells must be seeded directly inside the bioreactor, making it difficult to control the exact culture area and to monitor cell viability during the assay. Furthermore, any alterations during the cell culture may compromise the function of the device, which cannot be re-used.

In our work, a bioreactor integrating a modified commercial membrane that separates two cell culture chambers is proposed to facilitate the study of the liver sinusoid. In particular, this bioreactor simulates the architecture and microcirculation in the liver in order to properly analyze the paracrine interactions between cells.

## Materials and Methods

### Concept and design of the bioreactor

**Figure 1:**
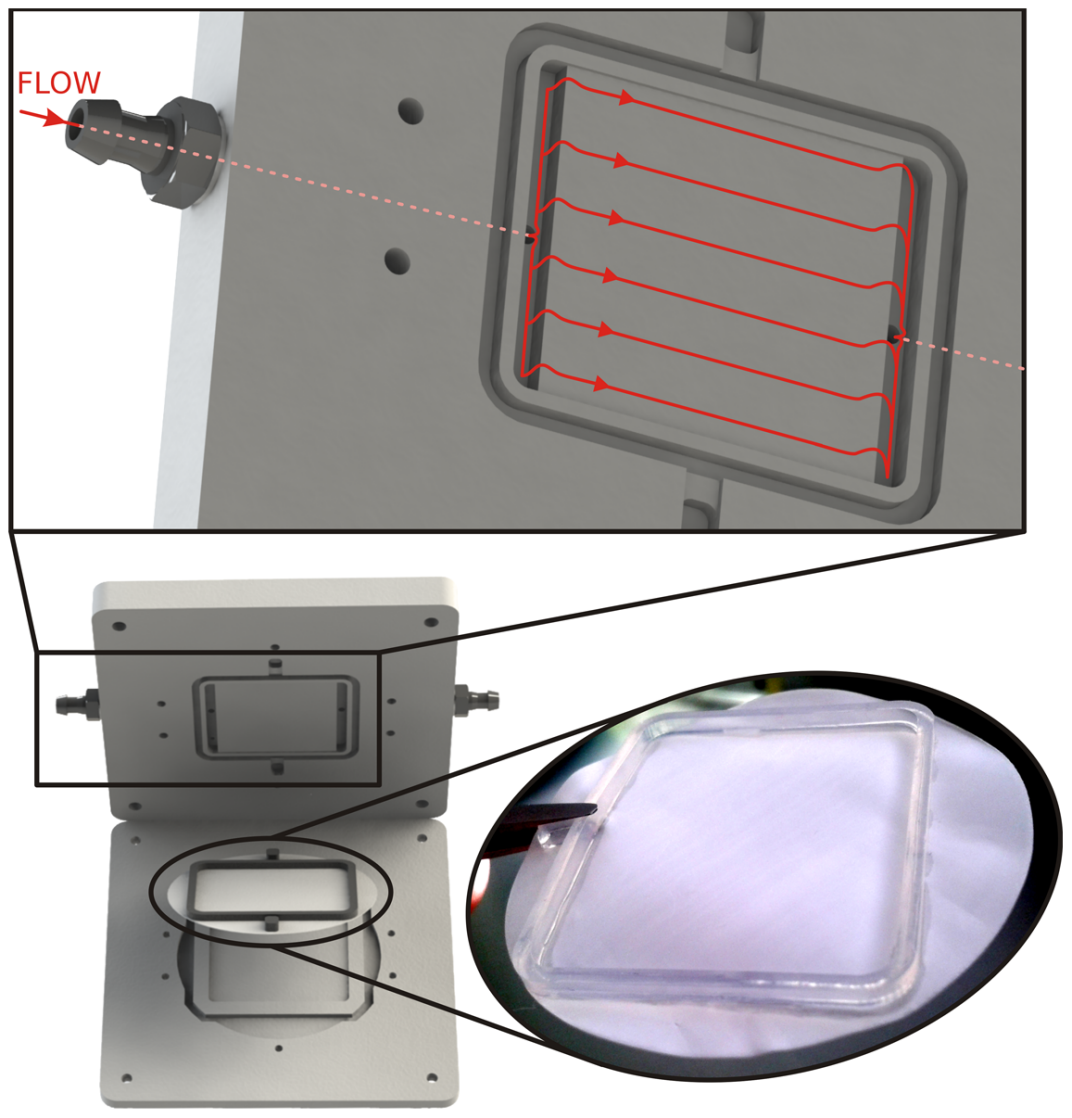
Schematic of the two-plate bioreactor integrating the home-modified membrane, with detail of the upper plate where the flow paths are shown in red (top).

This novel bioreactor is a modular system that comprises two transparent plates and a porous membrane that can be easily mounted and dismounted. To do that, the membrane is modified by adding an elastomeric wall (figure 1). The wall provides mechanical stability to the membrane, defines the cell culture area, and seals the assembly into the bioreactor. Then, it is possible to seed cells on the membrane outside the bioreactor, in static conditions following the well-known standards that are used in the laboratory.

The upper plate includes trenches where the elastomeric wall manufactured on the membrane will fit and a channel to perfuse the medium over the endothelial cell culture. To ensure a well-defined, laminar and homogeneous fluid flow along the whole channel area, and over the membrane, a parallel-plate flow chamber configuration was used [33]. In particular, small canals that connect the external microfluidic set-up with the inner channel fall into a couple of reservoirs that are placed along the width of the channel. While the cells placed below these reservoirs would not be under uniform flow conditions, it can be assumed that more than the 95% of the area of the channel is under the same flow conditions [34]. Therefore, the shear stress sensed by the cells cultured on the membrane can be assumed to be uniform.

The lower plate of the bioreactor contains a rectangular pool with no fluidic connection where a second cell type can be seeded while being protected from the shear stress during the cell-culture. This pool has the same area as that delimited by the elastomeric wall on the membrane. Moreover, an external pool was also designed to receive the excess of liquid from the inner pool once the bioreactor is closed.

### Fabrication

The present bioreactor aims to include a culture area large enough to allow optimal cell seeding and culture, along with multipurpose, post-experimental analysis. Although some molecular studies can be done in a single-cell, most biomedical research requires a decent amount of cells to cooperate and communicate such that a large range of molecular assays can be performed. For that, commercial hydrophilic microporous membrane filters (47 mm in diameter, 100 um thick, and 1 µm of pore size, Omnipore, Millipore, USA) were chosen.

To manufacture the 2 mm×1.5 mm elastomeric wall on the membrane encompassing an area of 969 mm^2^ (34×28.5 mm), a mould with the negative of the wall-structure was fabricated in Teflon by CNC machining. Next, the mold was filled with a UV-curable silicon-based elastomeric adhesive (Loctite 5055, Henkel). Then, the membrane was placed between the mold and a polymethilmetacrylate (PMMA) cover. After a few seconds, the full-sandwiched system was placed under a UV Curing Light Lamp System (PC-5000, Dymax) with irradiance of 62 mW cm^−2^ at the wavelength of 365 nm for 60 s in order to cure the silicone adhesive. Finally, the membrane attached with the cured elastomeric wall was gently peeled off from the mold.

The fabrication of the bioreactor was performed by CNC machining on a 5 mm (for the lower plate) and 8 mm thick (for the top plate where microfluidic connections are included) PMMA plates. In particular, the cell-culture pool on the lower plate had a depth of 1 mm, a distance fully adequate for paracrine interactions since it is much smaller than commonly used cell culture inserts. Meanwhile, the perfusion channel micromachined in the upper plate was 500 µm deep.

In order to place the second cell type on the top of the lower plate, the PMMA surface needs to be modified [35]. For that, hydrophilization by means of a corona discharge treatment was applied to it. The same treatment was applied to the bottom of the upper plate to facilitate fluid flow and to minimize the generation of bubbles that can occur on hydrophobic surfaces.

### Cell culture

The hepatic sinusoid was mimicked using endothelial cells and hepatic stellate cells previously validated as gold standard methods for vascular and hepatic research [36], [37]. Primary human umbilical vein endothelial cells were plated at a confluency of 85% on the gelatin-coated membrane. Human activated hepatic stellate cells (LX-2) were plated at a confluency of 80% on the lower plate. Cells were separately seeded and cultured until assembly of the bioreactor. On the day of the experiment, cells exhibited 95% confluency.

### Assembly and perfusion

**Figure 2:**
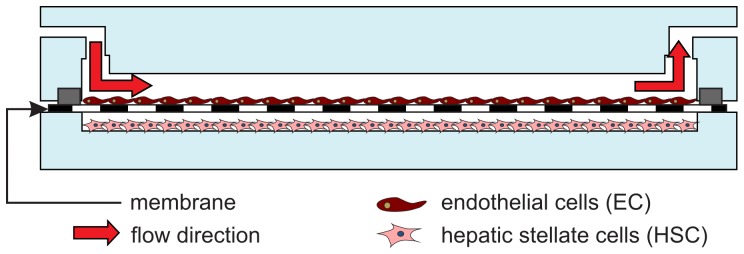
Cross-section schematic of the mounted bioreactor with the endothelial cells culture on top of the home-modified membrane and the HSC culture on top of the lower plate.

The bioreactor was assembled, as shown in figure 2, and connected to the perfusion system using barbed connectors. Silicone tubing was used to connect the recirculating microfluidic set-up with a commercial peristaltic pump (Minipuls 3, Gilson). The pump injects the perfusion media (M199 medium with 10% FBS, 100 U/mLPen/Strep, 2 mM Glutamine, and 2% Dextran) from a flask to the bioreactor. A commercial, 5 µm pore-size membrane filter with an integrated venting system (Speedflow Kids, GVS) was placed in front of the bioreactor inlet to guarantee total safety against air bubbles.

In the current set-up, the peristaltic pump was placed outside the incubator to preserve its integrity. Then, the perfusion media was held at ambient conditions on its way from the flask to the bioreactor. This avoids bubble generation, especially if the perfusion media is stirred during preparation. To further avoid bubble generation problems, the media was degasified in a vacuum chamber before use and left in an incubator overnight. Once the media was stabilized under the desired conditions, (37°C, 5% CO_2_) the full system was connected and the peristaltic pump switched on.

The fluid flow through the channel above the membrane where the endothelial cells were cultured was chosen taking into account the formula for the shear stress *τ* via perfusion in a parallel-plate flow chamber [38]:
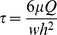
where *μ* is the media viscosity, *Q* is the flow rate and *w* and *h* are the channel width and height respectively. In this study, a shear stress sufficient to stimulate the endothelial population of 3 dyn/cm^2^ was applied for 24 h [39]–[41]. The bioreactor was then disassembled and the two cell types were separated and analyzed individually. By design, the hepatic stellate cells on the lower plate were cultured under static conditions, as it happens in the hepatic sinusoid.

### Cells phenotype analysis

Endothelial cell morphology and the production of nitric oxide were analyzed in real time by membrane and nuclei staining using Image-IT kit LIVE (Invitrogen), and by staining with DAF-FM (Invitrogen), respectively. Nuclei and membrane staining is necessary to evaluate the morphological state, the bonding and the alignment of the cells after shear stress stimulation. Similarly, the production of nitric oxide (NO) represents an excellent functional test to validate the improvement of the endothelial phenotype due to biomechanical stimulus (shear stress). Measurement of NO in real time has been described in depth elsewhere [42], [43]. Briefly, cells are washed with PBS, incubated 20 min with phenol red-free medium with 10 uM DAF-FM, and photographed using a fluorescent microscope equipped with a digital camera (Olympus BX51). 10 pictures per experimental condition are analyzed using ImageJ software (NIH). In each experiment, a control condition was cultured in static conditions in the same incubator. Specificity controls for DAF-staining were included (cells co-incubated with the NO synthase inhibitor L-NAME 1.5 mM) [44].

For HSC phenotype characterization, two major markers of their phenotype, alpha-smooth muscle actin (α-SMA) and pro-collagen I, were analyzed using qPCR [37]. Previous work from this team and others validated the analysis of these two genes as a way to analyze the phenotype of HSC [37], [45].

## Results and Discussion

The bioreactor presented in this work improves the usability of the reported bioreactors. In particular, the elastomeric structure that is manufactured over a porous membrane facilitates the experimental procedure as it defines the cell culture area and provides significant mechanical robustness to often delicate membranes. Then, cell seeding over the membrane can be performed outside the bioreactor, allowing the use of well-established protocols under static conditions. This results in improved test reliability since the viability of the cell culture will no longer affect the outcome.

A final, but no less important, advantage of the device presented in this work is the possibility to separate cell types after the experiment. Since the cell cultures are placed on different substrates, they can easily be separated (membrane and lower plate), and tested independently as it was done in order to present the following results.

### Endothelial cells phenotype after culture within the bioreactor

**Figure 3:**
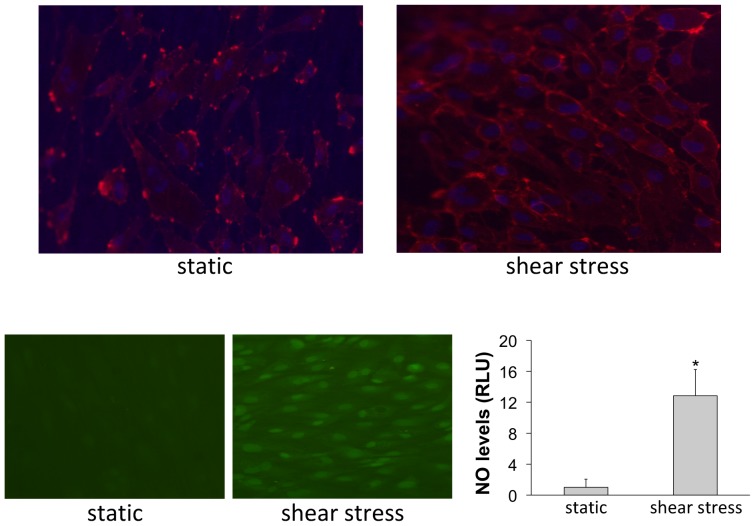
Representative images of endothelial cells cultured in the present system under continuous perfusion (shear stress) and static conditions (static). *Top*, Cell membrane staining (red) and nuclei (blue). *Bottom*, Real-time production of nitric oxide (green) and fluorescence quantification (data come from n = 3 experiments; and fluorescence intensity was divided by the total number of cultured cells; *p<0.05 vs. static t-test).

As shown in figure 3, endothelial cells cultured under shear stress stimulation maintained the confluence established before starting the shear stress and showed a correct morphology of a stretched cell, oriented in the direction of the flow. This is not observed in cells cultured under static conditions. Additionally, endothelial cells cultured under biomechanical stimulation showed markedly higher production of NO in comparison to those cells cultured in static conditions. This demonstrates that the application of shear stress markedly improves the stimulation of the endothelium. Although future experiments will validate the system using other magnitudes of shear stress, and/or, in other types of endothelial cells, the experiments herein reported demonstrate an optimal endothelial stimulation conferred by the system.

### Hepatic stellate cells phenotype after co-culture within the bioreactor

**Figure 4:**
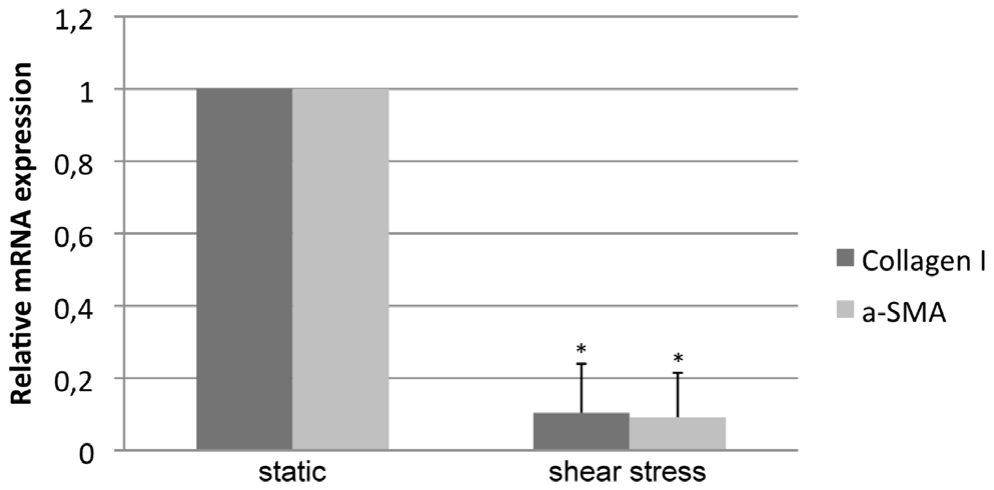
mRNA expression of two activation markers (a-SMA and Collagen I) in hepatic stellate cells co-cultured in the bioreactor with endothelial cells under shear stress stimulus or under static conditions. The reduction in both markers indicates an improvement in the stellate cells phenotype, most probably derived from the nitric oxide produced by the endothelial cells stimulated with shear stress (data from n = 3 experiments; * p<0.01 vs. static t-test).

Hepatic stellate cells co-cultured in the bioreactor with shear-stress stimulated endothelial cells exhibited a much lower expression of both phenotypic markers, α-SMA and pro-collagen I, than did cells co-cultured without biomechanical stimulation. This suggests that significant amelioration in the phenotype was due to paracrine signaling from stimulated endothelial cells (Figure 4). Considering our previous report demonstrating the amelioration of HSC phenotype due to paracrine release of NO from simvastatin-treated endothelial cells [37], we propose a shear stress-derived endothelial NO-dependent mechanism to explain the improvement in HSC status.

Figure 4 is particularly relevant as it demonstrates that the bioreactor presented here allows paracrine interactions between co-cultured cells. This is a significant improvement over the previous method of using common cell culture inserts under static conditions since our bioreactor simulates the *in vivo* situation of the hepatic vasculature. The improved communication of hepatic cells and the modification in their phenotype validate the bioreactor design described here. Indeed, the effects of endothelial cells on hepatic stellate cells phenotype in a physiological-like environment is much prominent than those observed using ordinary co-culture assays [37].

## Conclusions

The successful demonstration of the bioreactor described here enables improved study of the structure and function of the liver sinusoid. Permitting different cell types to be easily co-cultured under physiological conditions and separately analyzed afterwards simplifies and improves the reliability of clinical work on liver sinusoids. Importantly, our report demonstrates the improvement of hepatic stellate cells phenotype due to paracrine factors released from endothelial cells cultured under biomechanical stimulation. This has not been described in the literature to the best of our knowledge due to the unavailability of an easy-to-use bioreactor that allows cell co-culture under uniform and controlled shear stress stimulation.

In addition, these successful results point out the feasibility of using the proposed bioreactor for basic *organ on a chip* experimentation, as different cell types can be easily co-cultured in an *in vivo* environment. This is because of the excellent control of the seeding processes and the post-experimentation analysis possibilities; therefore, the applicability of this bioreactor in other vascular systems different from the liver is promising but will require further study.
